# Feasibility Study of Virtual Reality–Based Cognitive Behavioral Therapy for Patients With Depression: Protocol for an Open Trial and Therapeutic Intervention

**DOI:** 10.2196/49698

**Published:** 2023-09-26

**Authors:** Ai Ito, Fumikazu Hiyoshi, Ayako Kanie, Azumi Maruyama, Mari S Oba, Shinsuke Kito

**Affiliations:** 1 Department of Clinical Psychology National Center Hospital National Center of Neurology and Psychiatry Kodaira-shi Japan; 2 Brain Bioregulatory Science Cooperative Graduate School The Jikei University Graduate School of Medicine Minato-ku Japan; 3 Jolly Good Inc Chuo-ku Japan; 4 Department of Child Neropsyhchitatry The University of Tokyo Hosoital Bunkyo-ku Japan; 5 National Center for Cognitive Behavior Therapy and Research National Center Hospital National Center of Neurology and Psychiatry Kodaira-shi Japan; 6 Department of Clinical Data Science Clinical Research and Education Premotion Division, National Center Hospital National Center of Neurology and Psychiatry Kodaira-shi Japan; 7 Neuromodulation Therapy and Research Center National Center Hospital National Center of Neurology and Psychiatry Kodaira-shi Japan

**Keywords:** depression, cognitive behavioral therapy, virtual reality, CBT, feasibility study, open trial, VR

## Abstract

**Background:**

The clinical usefulness of cognitive behavioral therapy (CBT) for patients with depression who do not remit with pharmacotherapy has been recognized. However, the longer time burden on health care providers associated with conducting CBT and the lack of a system for providing CBT lead to inadequate CBT provision to patients who wish to receive it.

**Objective:**

We aim to evaluate the feasibility of introducing virtual reality (VR) into CBT for patients with depression.

**Methods:**

This is a single-center, interventional, exploratory, single-arm, nonrandomized, open, pre-post–comparative feasibility study of an unapproved medical device program to evaluate the acceptability, preliminary efficacy, and safety of the study device. Eligible patients meet the diagnostic criteria of the DSM-5 (Diagnostic and Statistical Manual of Mental Disorders, 5th Edition) for major depressive disorder, have a 17-item Hamilton Depression Rating Scale (HAMD-17) score of ≥12, and are aged 18-65 years. The sample will comprise 12 patients. VR-based CBT (CBT-VR) sessions will be conducted once a week in an outpatient setting. CBT-VR has been developed in accordance with 6 stages and 16 sessions in the current CBT therapist manual. VR contents and other components correspond to the themes of these 16 sessions. The flow of CBT-VR treatment is similar to that of normal CBT; however, this product replaces the in-person portion of CBT. The primary end point will be the change in the HAMD-17 score from baseline up to 16 sessions. Secondary end points will be treatment retention; psychiatrist consultation time; satisfaction with the equipment or program; ease of use; homework compliance; change in the HAMD-17 score from baseline up to 8 sessions; change in Montgomery-Åsberg Depression Rating Scale (MADRS), Quick Inventory of Depressive Symptomatology Self-Report (QIDS-SR), EQ-5D-5L, and Clinical Global Impressions (CGI) scores from baseline up to 8 and 16 sessions; and change in remission and response rates and HAMD-17, MADRS, QIDS-SR, and EQ-5D-5L scores from baseline to 3 and 6 months post intervention (or discontinuation). CBT-VR’s feasibility will be assessed at baseline, after 8 sessions, after 16 sessions, or treatment discontinuation, by measuring the time required for testing and medical care during each session and with a patient questionnaire. After intervention discontinuation, a follow-up evaluation will be conducted unless the patient withdraws consent or otherwise discontinues participation in the study after 3 and 6 months.

**Results:**

Participant recruitment started on November 30, 2022, and data collection is ongoing as of September 2023.

**Conclusions:**

This study is the first step in testing the acceptability, feasibility, and preliminary efficacy and safety of CBT-VR for patients with depression without controls in an open-label trial. If its feasibility for depression treatment is confirmed, we intend to proceed to a large-scale validation study.

**Trial Registration:**

Japan Registry of Clinical Trials jRCTs032220481; https://jrct.niph.go.jp/en-latest-detail/jRCTs032220481

**International Registered Report Identifier (IRRID):**

DERR1-10.2196/49698

## Introduction

Depression is a common disorder, with a lifetime prevalence rate of over 10% [1,2].

It is generally treated with antidepressant medication. However, some patients with depression do not respond to those medications, and depressive symptoms may persist for a long period without adequate improvement [3], resulting in a significant emotional and financial burden on the patients and their families [4-6].

Cognitive behavioral therapy (CBT) is a structured psychotherapy that modifies cognitive biases and assists problem-solving since mood is influenced by how one perceives and acts. CBT comprises several components: cognitive restructuring, behavioral activation, assertion, problem-solving, and relaxation [7,8]. CBT for patients with depression consists of 16-20 sessions of at least 30 minutes each, mainly through face-to-face sessions.

The efficacy of CBT for patients with depression who do not remit with pharmacotherapy has been tested in a randomized controlled trial in Japan [9]. Eighty patients with drug-resistant major depressive disorder were randomized to CBT or no-CBT groups. The change in the 17-item Hamilton Depression Rating Scale (HAMD-17) score from baseline to 16 weeks later was compared, with a significant improvement in the HAMD-17 score in the CBT group compared to that in the no-CBT group (between-group difference: –5.4 points; *P*<.001). Although the clinical usefulness of CBT has been recognized and insurance coverage is available when performed by a physician or nurse, the provision of CBT to patients who wish to receive it is currently inadequate. This can be attributed to the prolonged time burden on health care providers when performing CBT and the lack of a system to provide CBT. Therefore, developing a delivery system that uses information and communication technology is a challenge that must be overcome to enable the widespread use of CBT.

Web-based CBT has the same content as face-to-face CBT; however, it is more flexible and cost-effective treatment than face-to-face therapy for depression. Web-based CBT significantly improved patients’ depressive symptoms and quality of life, similar to face-to-face CBT [10]. Mixed treatment integrating face-to-face CBT and web-based CBT has also been shown to be effective in treating patients with depression [11]. CBT can also be delivered through virtual reality (VR), a technology that immerses users in a computer-generated virtual world [12]. VR is considered a powerful educational tool because it allows people to experience, rather than merely perceive, what they need to learn [13].

However, several previous studies of VR interventions for mental health have focused on treating anxiety disorders [12,14], pain distraction [15,16], and relaxation [17,18], with surprisingly few VR interventions for depression [19].

In a joint study conducted in 2020 by the National Center for Cognitive Behavioral Therapy, Jolly Good Inc, and the National Center of Neurology and Psychiatry, the impact on depressive symptoms and safety of VR technology in providing CBT for patients with depression were evaluated (University hospital Medical Information Network [UMIN] Clinical Trials Registry; UMIN000040864). As a result, 4 of 7 patients included in the study had improvements in 2 or more Beck's Depression Inventory-II depression rating scale severity categories. One of 7 patients showed an improvement in 1 severity category compared to the previous treatment. No adverse events were observed, suggesting that using VR technology during CBT for patients with depression is well tolerated. However, this previous study has yet to be published. This previous study aimed to increase positive emotions in 7 patients with depression. The VR content comprised images of landscapes, animals, and a woman laughing, but not the CBT content.

The newly developed VR-based CBT (CBT-VR) uses VR to deliver CBT content to patients with treatment-resistant depression. CBT-VR for depression in this study aims to help patients acquire CBT skills, become aware of their own automatic thought patterns through the examples of others, and guide them toward solutions. Therefore, the VR content in this study is different from that in the previous study.

The newly developed CBT-VR follows the 16-session agenda in the therapist’s manual and is intended to treat patients with depression in the same way as current CBT. CBT-VR can replace a part of the CBT conducted face-to-face by a health care provider and reduce the longer time burden. It is expected to expand opportunities for the treatment of depression and improve patient benefits through CBT.

In this study, we will evaluate whether it is possible to reduce the intervention time of medical personnel while retaining the effects of CBT by replacing part of the CBT conducted in person with VR and videoconferencing, using information terminals. By incorporating VR into CBT, patients can learn CBT skills and become aware of their own automatic thought patterns through the examples of others. Changing one’s mindset is important in CBT for depression, and the advantage of VR is that it provides an immersive experience that puts one in another person's shoes. The CBT-VR system to be used in the study is an unapproved medical device program under development by Jolly Good Inc, which intends to apply for the relevant manufacturing and marketing approvals in collaboration with Teijin Pharma Limited.

This study aims to evaluate the feasibility of introducing VR into CBT for patients with depression. In this study, 16 sessions of CBT-VR will be conducted, its acceptability will be assessed, and its efficacy and safety will be preliminarily examined by measuring and collecting data on the participants' usability and the time required for psychiatrist consultation.

If the feasibility of CBT-VR for depression is demonstrated in this study, we may proceed to a confirmatory study. In addition, if further work shows that CBT-VR is effective, it may provide a new option for patients who do not respond adequately to conventional treatment and have not had the opportunity to receive CBT.

## Methods

### Study Design

This is a single-center, interventional, exploratory, single-arm, nonrandomized, open, pre-post–comparative study involving a feasibility study of an unapproved CBT-VR device intervention to evaluate its acceptability, preliminary efficacy, and safety among patients with depression.

### Participant Selection Criteria

#### Inclusion Criteria

Patients who meet all the following criteria will be enrolled in the study: (1) those who meet the DSM-5 (Diagnostic and Statistical Manual of Mental Disorders, 5th Edition) diagnostic criteria for major depressive disorder; (2) those with a HAMD-17 score of ≥12 at screening; (3) those who do not respond adequately to ≥1 antidepressants or are unsuitable for pharmacotherapy due to intolerance, comorbidity, pregnancy, or refusal of medication; (4) those who are aged between 18 and 65 years when consent is obtained; and (5) those who provide written informed consent.

#### Exclusion Criteria

Patients meeting the following criteria will be excluded from the study: (1) those with comorbid or preexisting manic, hypomanic, or psychotic episodes; personality disorders or posttraumatic stress disorder; or alcohol or substance use disorders; (2) those with active suicidal ideation; (3) those who have not responded to CBT in the past; (4) those who have difficulty using VR devices and applications; and (5) those deemed inappropriate for this study by the principal investigator.

### Sample Size Determination

The sample size will be 12 patients. This sample size was not determined on the basis of the distribution of any end point or statistical power.

In order to evaluate the acceptability of CBT-VR in this study, it is necessary to obtain data on use and satisfaction from a wide range of patients (based on parameters such as sex, age, and severity). In addition, a certain number of patients is necessary to evaluate efficacy and safety. A similar pre- and postintervention comparative study of CBT using VR for patients with depression (UMIN000040864) was conducted with 12 patients, and the number of patients was deemed to be sufficient for feasibility evaluation.

### Study Outline

Screening tests will be performed for patients who have obtained consent to participate in the study, and baseline testing will be performed for eligible participants before the start of the intervention. In principle, 16 CBT-VR sessions will be administered weekly in an outpatient setting. The feasibility of CBT-VR will be assessed by testing at baseline, at the end of 8 sessions, at the end of 16 sessions, or treatment discontinuation, and by measuring the time required for testing and medical care during each session and by patient questionnaire. The duration of the intervention, including allowance, will be a maximum of 24 weeks, with no interruption of more than 8 consecutive weeks from the end of each session to the implementation of the next session. In addition, no more than 1 session will be conducted on the same day. If a patient is hospitalized during the intervention period, participation in the study will not be discontinued if it is possible to continue. After the intervention is terminated, follow-up assessments will be conducted unless the patient withdraws consent or otherwise discontinues participation in the study. After 16 sessions or after the intervention is terminated, patients will be treated as usual; however, they will continue to view the VR and use the application for 6 months during the follow-up period.

### Information on Drugs, Medical Devices, and Treatments to be Used in the Study

A CBT-VR device (Jolly Good Inc) will be used in this study. CBT-VR is an unapproved medical device program developed to support CBT for patients with depression.

The system structure of this product is shown in Table 1. This product is a system that integrates 6 components consisting of VR contents installed on general-purpose commercial VR goggles, 3 applications used by patients or health care providers, and progression support tools with CBT-VR content management system in the cloud.

CBT-VR has been developed in accordance with 6 stages and 16 sessions described in the current CBT therapist manual, and the VR contents and other components correspond to the themes of each of these 16 sessions. The treatment flow using CBT-VR is similar to that of the normal CBT protocol; however, this product replaces a part of the CBT conducted face-to-face by a health care provider with VR and videoconferencing. The homework app will be downloaded to the patients’ smartphone.

Adverse events anticipated from CBT-VR include pain in the area where the VR goggles are worn and discomfort from viewing the VR images (VR sickness), both of which are expected to be mild and tolerable.

**Table 1:** List of names and functions of each component, equipment, and users.

Name	Main functions	Equipment used	User
Viewer application	Content playback, choice operation, and choice data acquisition	VR^a^ goggles	Patient
Homework app	Homework implementation and scheduling	Smartphone	Patient
Manager application	Content control	Tablet terminal	Health care provider
My Page	Patient information registration and data viewing	PC or tablet device	Health care provider
VR contents	Video and audio of CBT^b^ sessions	VR goggles	Patient
Progression support tools	Manual for health care providers and manual for patients	Tablet terminal or printed matter	Health care provider and patient
CBT-VR^c^ CMS^d^	System integration of VR goggles, the Viewer application, the Homework app, the Manager application, My Page, and Progression support tools	Cloud server	Jolly Good Inc

^a^VR: virtual reality.

^b^CBT: cognitive behavioral therapy.

^c^CBT-VR: virtual reality–based cognitive behavioral therapy.

^d^CMS: content management system.

### Dosage and Administration of Drugs, Applications, or Equipment Used in This Study

In this study, 16 sessions of CBT-VR will be conducted once a week in an outpatient setting. The flow of each session of CBT using this product is described in Table 2.

When participants arrive at the clinic, they will review their homework using the progression support tool and experience the theme by viewing the VR content corresponding to each session. While using the progression support tool again, they will use the VR experience as a guide to practice the themes. The participant will then interact with the physician and conduct homework at home using the Homework App and the progression support tool [20,21].

If depressive symptoms exacerbate and require hospitalization after each session, the principal investigator will decide whether or not to continue the treatment.

**Table 2:** The flow of each session.

Session	Progress of the session	Components	Implementor
1	Introduction	Progression support tools	Patient
2	Theme experience	VR^a^ goggles (includes content and other programs)	Patient
3	Theme practice	Progression support tools	Patient
4	Dialogue on the theme (medical treatment by a physician)	Progression support tools	Physicians and patients
5	Homework (conducted at home)	Homework app and Progression support tools	Patient

^a^VR: virtual reality.

### Rules for Concomitant Medications and Concomitant Therapy

The type and dosage of concomitant antidepressants will not be changed during the intervention period from baseline to the examination at the end of the 16 sessions or at the end of the intervention. Dosage and administration will be in accordance with the package insert of each antidepressant. If no concomitant antidepressants are used, no new antidepressants will be initiated during the intervention period. No structured psychotherapy, repetitive transcranial magnetic stimulation, or electroconvulsive therapy will be administered during the intervention period, and treatment will not be restricted during the follow-up period after 16 sessions or after discontinuation of the intervention.

### End Points and Evaluation Methods

The primary end point is a change in the HAMD-17 score from baseline to the end of 16 sessions. The secondary end points are the treatment continuation rate (ie, the proportion of participants who completed 16 sessions); the time required for psychiatrist consultation; patient satisfaction and usability of the equipment and programs and compliance with homework; change in the HAMD-17 score from baseline to the end of 8 sessions; change in the Montgomery-Åsberg Depression Rating Scale (MADRS), Quick Inventory of Depressive Symptomatology Self-Report (QIDS-SR), EQ-5D-5L, and Clinical Global Impressions (CGI) scores from baseline to the end of 8 and 16 sessions; remission rates (ie, the proportion of participants with a HAMD-17 score of ≤7 and a <ADRS score of ≤10); response rates (ie, the proportion of participants with a ≥50% reduction in the HAMD-17 and MADRS scores from baseline); change in HAMD-17, MADRS, QIDS-SR, EQ-5D-5L, and CGI scores from baseline to 3 and 6 months post intervention (or discontinuation); the proportion of participants with a decrease in the number of prescriptions or doses of psychotropic drugs from baseline to 3 and 6 months post intervention (or discontinuation); adverse events; and equipment failures.

For the safety evaluation, pain, discomfort from wearing or viewing VR, manic and hypomanic episodes (meeting the DSM-5 criteria), and suicidal ideation will be assessed during the intervention and observation periods. Suicidal ideation will be assessed using HAMD-17, MADRS subitems, and clinically. The interviewer will check for the presence and worsening of suicidal ideation at each session.

### Observation and Examination Items

The following items will be collected per schedule: patient background (age, sex, height, weight, comorbidities, medical history, educational history, employment experience, and VR viewing experience); depression treatment history (date of diagnosis, number of previous episodes, duration of the current episode, number of antidepressant failures due to Antidepressant Treatment History Form, and history of CBT treatment); concomitant therapy (including medications, repetitive transcranial magnetic stimulation, and electroconvulsive therapy, and whether they were hospitalized); satisfaction with CBT-VR, feeling of use, and compliance with homework; time required for consultation by health care providers; HAMD-17, MADRS, QIDS-SR, EQ-5D-5L, and CGI scores; adverse events; and equipment failures.

**Figure 1:**
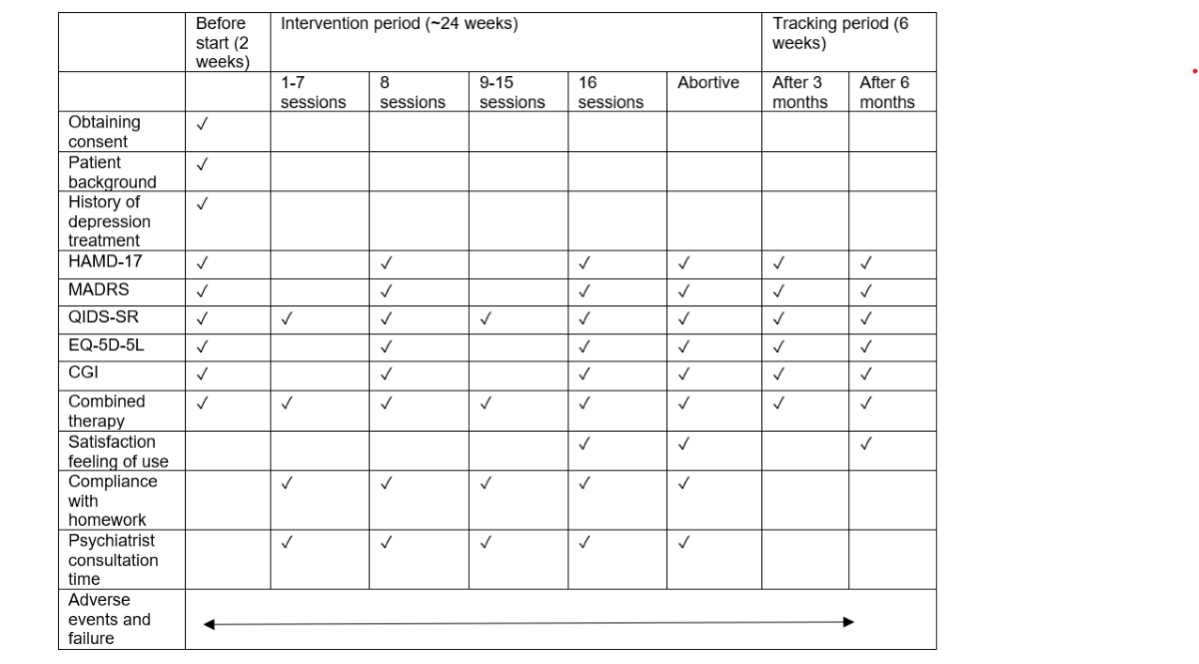
Observation and inspection schedule. Combined therapy during the 6-week tracking period after 3 and 6 months post intervention include continuous use of VR or applications. CGI: Clinical Global Impressions; HAMD-17: 17-item Hamilton Depression Rating Scale; MADRS: Montgomery-Åsberg Depression Rating Scale; QIDS-SR: Quick Inventory of Depressive Symptomatology Self-Report; VR: virtual reality.

The observation schedule is shown in Figure 1. In total, 16 CBT-VR sessions will be performed weekly in an outpatient setting. The intervention period will be a maximum of 24 weeks, including allowance, with no interruption of more than 8 consecutive weeks between the end of each session and the implementation of the next session. No more than 1 session will be conducted on the same day. The day of the week will not be fixed, and only 1 session will be carried out per week. If a session is held on Friday, the next session will be held on any day from Monday to Friday the following week. Therefore, the minimum interval between sessions will be 2 days (Friday to Monday).

### Statistical Analysis

The study objective is purely descriptive. We do not intend to decide the efficacy and safety of CBT-VR based on statistical tests.

#### Population for Efficacy and Safety Analysis

The population for efficacy analysis includes all participants who have used CBT-VR at least once and at least 1 end point is measured. The population for safety analysis consists of all participants who have viewed CBT-VR at least once.

#### Analyses

Baseline characteristics will be assessed using descriptive statistics.

As a primary analysis, original scores and changes in the score on the HAMD-17 from baseline to the end of 16 sessions will be summarized, the latter depicted as mean and 95% CI values. A 1-sample *t* test against the null hypothesis that the mean change in score equals 0 will be performed. The threshold for statistical significance will be set at 5%, and multiplicity will not be adjusted since this study is exploratory by nature. Missing data will be imputed using the previous measurement.

As secondary analyses, original scores and changes in the scores on the HAMD-17 from baseline to other time points will be summarized, and mean and 95% CI values will be estimated. The same analyses will be performed for changes in MADRS, QIDS-SR, EQ-5D-5L, and CGI scores. The frequency and proportion of the patients in remission at each measured point and those with a decreased number or dose of psychotropic medications will be summarized. The frequencies and proportions of the patients who completed the 16 sessions, patient satisfaction, and usability (the Japanese version of Client Satisfaction Questionnaire-8 and other answers to questions) will be summarized for feasibility assessment. The time required for psychiatric consultation and the number of homework assignments done will be tabulated and summarized for each session. In addition, missing data will be excluded from the analysis at each measured point.

Detailed definitions for classification will be determined on the basis of the distribution before data fixation. The stratification variables for subgroup analyses are as follows: sex, duration of the current episode (<2 years and ≥2 years), age, work experience, previous CBT treatment, baseline HAMD-17 score, concomitant medications, homework compliance, time spent at the doctor’s office, continued viewing of VR content and use of applications (during follow-up).

As a safety analysis, the frequency of adverse events and failures will be tabulated for each event.

### Ethical Considerations

The clinical study will be conducted in compliance with the Clinical Trials Act. It was registered with the Japan Registry of Clinical Trials (trial ID jRCTs032220481) and approved by the Japan Certified Clinical Research Review Board. The study’s details will be fully explained to all patients, and face-to-face informed consent will be obtained before starting the study. After obtaining informed consent, the participants’ data will be deidentified before analysis.

If the obtained data are used in the future for purposes other than those indicated in this study, the following conditions will need to be met: (1) the study will be reviewed again and approved by the center's ethics committee or clinical research review committee, and (2) the willingness of the participants to cooperate in the study will be confirmed through reconsent or information disclosure regarding the conduct of another study.

## Results

This study was registered with the Japan Registry of Clinical Trials (trial ID jRCTs032220481). Informed consent will be obtained before enrollment in the study. Participant recruitment started on November 30, 2022, and data collection is ongoing as of September 2023. The results will be published in a peer-reviewed academic journal, presented at scientific conferences, and communicated to the participants and patient organizations. International Committee of Medical Journal Editors criteria on contributorship and authorship are applied.

## Discussion

This study is a first step toward testing the acceptability, feasibility, and preliminary efficacy and safety of introducing CBT-VR to patients with depression.

The efficacy of CBT for patients with depression who do not remit with pharmacotherapy has been verified in randomized controlled trials in Japan [3]. However, the provision of CBT to patients who wish to receive it is currently inadequate due to the large time burden on health care providers when conducting CBT and the lack of a system to provide CBT. In this study, we will evaluate whether it is possible to reduce the intervention time of health care providers while maintaining the therapeutic effects of CBT by replacing a portion of CBT conducted in person with VR and videoconferencing, using information terminals.

If this study confirms the feasibility of CBT-VR for depression, we may proceed to a confirmatory trial. Furthermore, if a future trial confirms that CBT-VR is effective, it may provide a new option for patients who have not had an adequate response to conventional treatment and have not had the opportunity to receive CBT.

As a limitation, the study will have a small number of cases, is not blinded, and will have no controls. In a future study, we would like to conduct a randomized controlled trial with appropriate controls.

Treatment options for CBT are expanding to include face-to-face, web-based, and VR components. Future developments may lead to new techniques, increasing access to CBT for more patients.

## Abbreviations

CBT: cognitive behavioral therapy
CBT-VR: virtual reality–based cognitive behavioral therapy
CGI: Clinical Global Impressions
DSM-5: Diagnostic and Statistical Manual of Mental Disorders, 5th Edition
HAMD-17: 17-item Hamilton Depression Rating Scale
MADRS: Montgomery-Åsberg Depression Rating Scale
QIDS-SR: Quick Inventory of Depressive Symptomatology Self-Report
UMIN: University Hospital Medical Information Network
VR: virtual reality

## Data Availability

The data sets generated or analyzed during this study will not be publicly available but will be available from the corresponding author upon reasonable request.
